# Announcement: Seventeenth Conference on Coordination Chemistry Coordination Chemistry at the Turn of the Century

**DOI:** 10.1155/MBD.1998.177

**Published:** 1998


### SEVENTEENTH CONFERENCE ON COORDINATION CHEMISTRY
### COORDINATION CHEMISTRY AT THE TURN OF THE CENTURY

Smolenice, Slovakia
7 - 11 June 1999

The conferences on coordination chemistry, held regularly in Slovakia, have a long tradition. The First Conference of this kind was organized in 1964. The Conferences took place in Smolenice Castle that was adapted for the needs of conferences and similar events and serves now as a Congress Centre of the Slovak Academy of Sciences. Many top coordination chemists from all over the world attended the Conferences till now and Smolenice Castle became the place where many new scientific and personal contacts were established.

The Organizing Committee invites you to participate to the 17th Conference on Coordination Chemistry. The Conference will be organized by the Slovak Chemical Society, Slovak Technical University and Comenius University in Bratislava and held in Smolenice Castle between 7 and 11 June, 1999. The Castle is situated in the middle of forests and parks on the southern slopes of the Low Carpathians. It is a wonderful place with a quiet atmosphere that creates ideal preconditions for fruitful and useful discussions.

As a rule, the weather in the region is very pleasant in the first decade of June, with temperatures of 20-25 °C. Since the Castle is about 60 km far from Bratislava, bus transport from Bratislava to Smolenice and retum will be organized on arrival and departure days, respectively.
Bratislava can be reached by plane, train, bus (from Vienna or Prague) or ship (from Vienna).

The official language of the Conference will be English.

**SCOPE OF THE CONFERENCE**

**A Structure and reactivity of coordination and organometallic compounds**
(Synthesis, structure, stability, kinetics, photochemistry, catalysis)

**B Supramolecular coordination chemistry**
(Solid state systems, host-guest chemistry, self-assembling compexes dendrimers, inorganic super structures, molecular modeling)

**C Complexes in biological systems, human medicine, and environment**
(Natural and model bio-complexes, structure-function relationship of therapeutic agents and enzymes, catalyzed destruction of pollutants)

**D Applied inorganic chemistry**
(Supramolecular devices, magnetic materials, superconductors, magnetic, electrical and optical properties of inorganic and coordination compounds, applications of inorganic catalysts)

### SCIENTIFIC PROGRAMME

The sclentific programme is supposed to be divided into four working days. Each of the topics will be introduced by a plenary lecture (45 minutes) delivered by an invited lecturer.
The other participants may present their contributions to the scientific programme as
- plenary lectures lasting 30 minutes, discussion included
- section lectures lasting 15 minutes, discussion included.
Young scientists are offered to use the possibility to present their results as posters.
However, each participant can deliver only one lecture/poster.

### CONTRIBUTED PAPERS

Each participant is expected to submit one paper only. One-page abstracts of all contributions will be published in the Proceedings of the 17th Conference on Coordination Chemistry. Those participants who wish present a 30 minutes lecture are required to submit their contribution as a six pages paper that will be published in a monograph entitled "Coordination Chemistry at the Turn of the Century" that will be printed by the Slovak Technical University Press in Bratislava. The papers will be reviewed by referees and their recommendations will be taken into consideration by the editors. The contributions of those who intend to present a poster will be published in the Proceedings only.
The participants will receive the Proceedings as well as the monograph together with reprints of their papers before the Conference at the registration.

### SOCIAL PROGRAMME

A social programme, including a welcome party and final banquet, is planned. Besides that, a half-day optional excursion (cultural and social event) in the surroundings of Smolenice will be organized.

### ACCOMMODATION

Accommodation for all participants will be arranged in Smolenice Castle. Since there are only 90 beds available for participants in the Castle the number of participants is obviously limited to this number. Consequently, those applicants will be preferred who show their interest to participate and send all required materials in due time.

### FEES

1) The registration fee is 150 USD.
This sum will cover all expenses connected with monograph, proceedings of the conference, reprints, conference materials, services.
2) TheConference fee is 150 USD.
It includes all expenses for board in Smolenice Castle (three complete meals per day, coffee & tea), social programme, services during the conference, travel expenses from Bratislava to Smolenice and retum
3) The lodging in Smolenice Castle will be paid separately. Thanks to the sponsorship of the Slovak Academy of Sciences, the sum to be paid for five nights shouhld not exceed 200 USD (depending on the category of rooms).

### PRELIMINARY APPLICATION

A Preliminary Application Form, that can be obtained from

Professor Gregor ONDREJOVIC
Department of Inorganic Chemistry
Slovak Technical University
Radlinského 9,
812 37 BRATISLAVA, Slovakia
Telephone: + 421 7 395 257
Fax: + 421 7 393 198
E-mail: sirota@cvtstu.cvt.stuba.sk should reach the Organizing Committee before September 30th, 1998.

### SECOND CIRCULAR

The Second Circular will be distributed at the end of October 1998.

